# Supplementary material for: Human A-to-I RNA editing SNP loci are enriched in GWAS signals for autoimmune diseases and under balancing selection
Source: Genome Biol. 2020 Nov 30;21:288. doi: 10.1186/s13059-020-02205-x (PMC7702712; doi:10.1186/s13059-020-02205-x)
Supplement: Supplementary file 1 — Additional file 1: Fig. S1. The pipeline of non-SNP and SNP editing site identification. Fig. S2. The identification of non-SNP and SNP editing sites. Fig. S3. Triplet motif analysis of SNP editing sites. Fig. S4. The verification of SNP editing sites. Fig. S5. Comparison of the ratios of RNA editing SNP and control SNP. Fig. S6. ADAR2 and CASQ2 expression in different types of human tissues. Fig. S7. The cumulative distribution of DAF. Fig. S8. Sliding window analysis of pi for A/G editing SNPs. Fig. S9. non-Alu editing SNPs are subject to a stronger balancing selection compared with Alu editing SNPs. Fig. S10. DAF analysis reveals RNA editing SNPs as the target of balancing selection in flies. [file 13059_2020_2205_MOESM1_ESM.pdf]

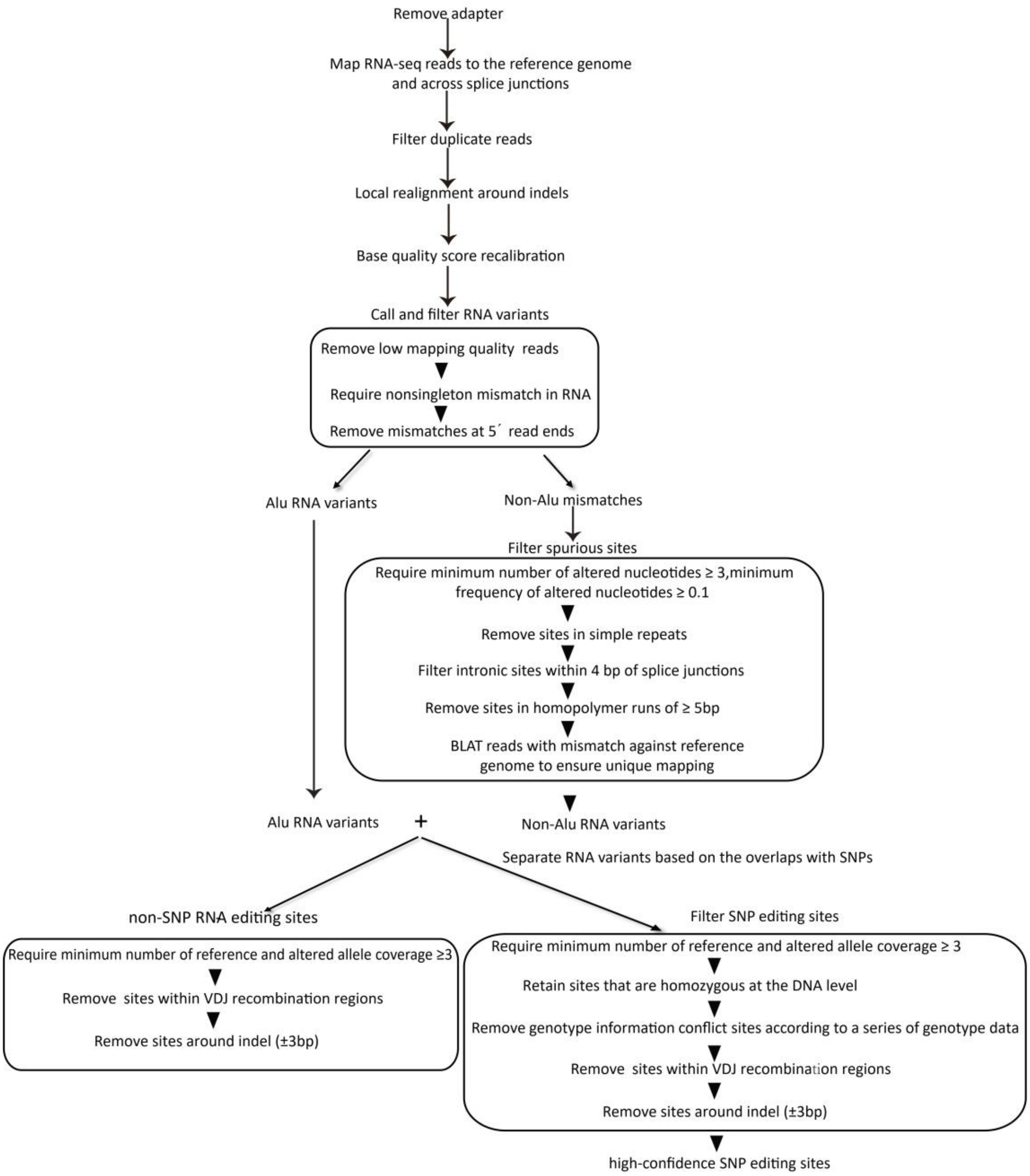

Fig. S1. The pipeline of non-SNP and SNP editing site identification.

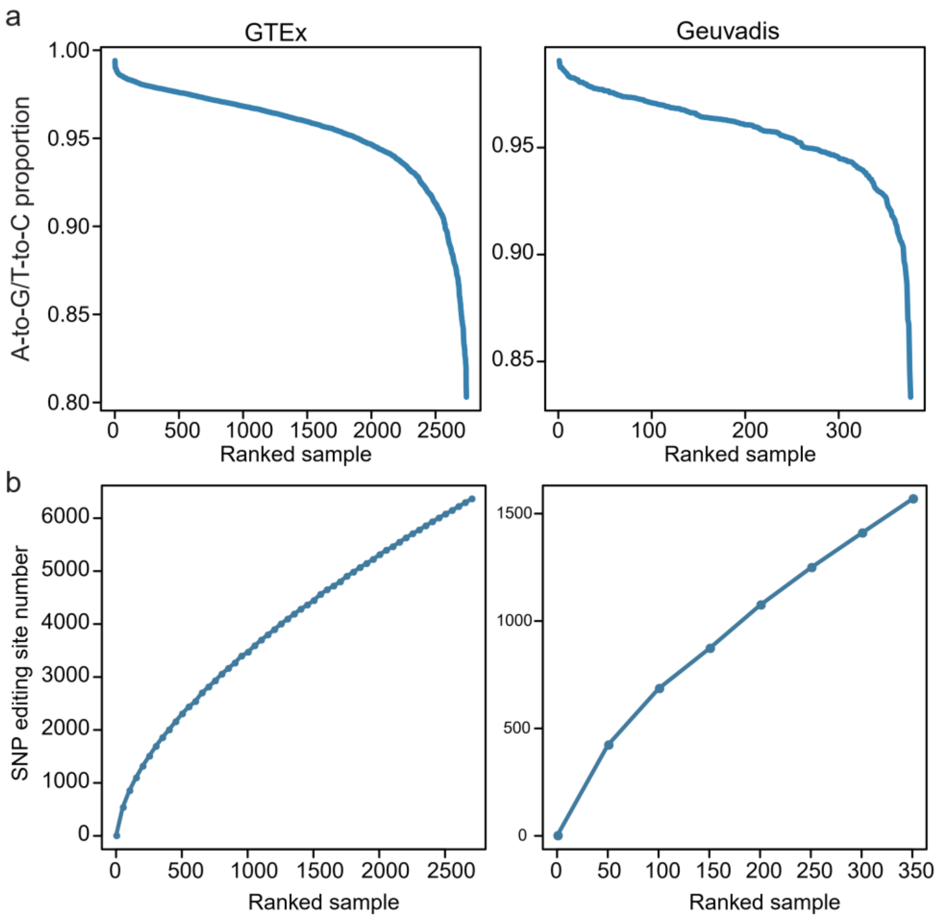

Fig. S2. The identification of non-SNP and SNP editing sites.

(**a**) The proportion of variants that are either A-to-G or T-to-C mismatches for non-SNP RNA variants in each sample. The GTEx and Geuvadis datasets were plotted, respectively.

(**b**) The relationship between the sample size and the number of A-to-G SNP editing sites identified. We carried out 100 independent random samplings of 50, 100, 150 … samples. Dots represent the mean of the SNP editing sites identified in each data point. The GTEx and Geuvadis datasets were plotted, respectively.

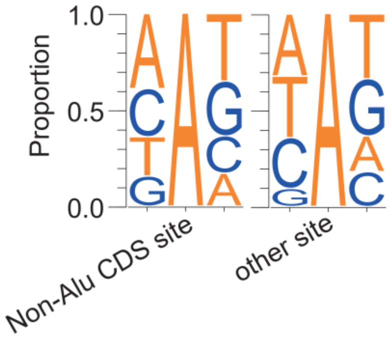

Fig. S3. Triplet motif analysis of SNP editing sites.

Because the Geuvadis dataset had a limited number of non-Alu CDS sites, we merged the SNP editing site lists from the GTEx and Geuvadis datasets for analysis. Non-Alu CDS sites (518 sites) and the remaining sites (7272 sites) were analyzed separately.

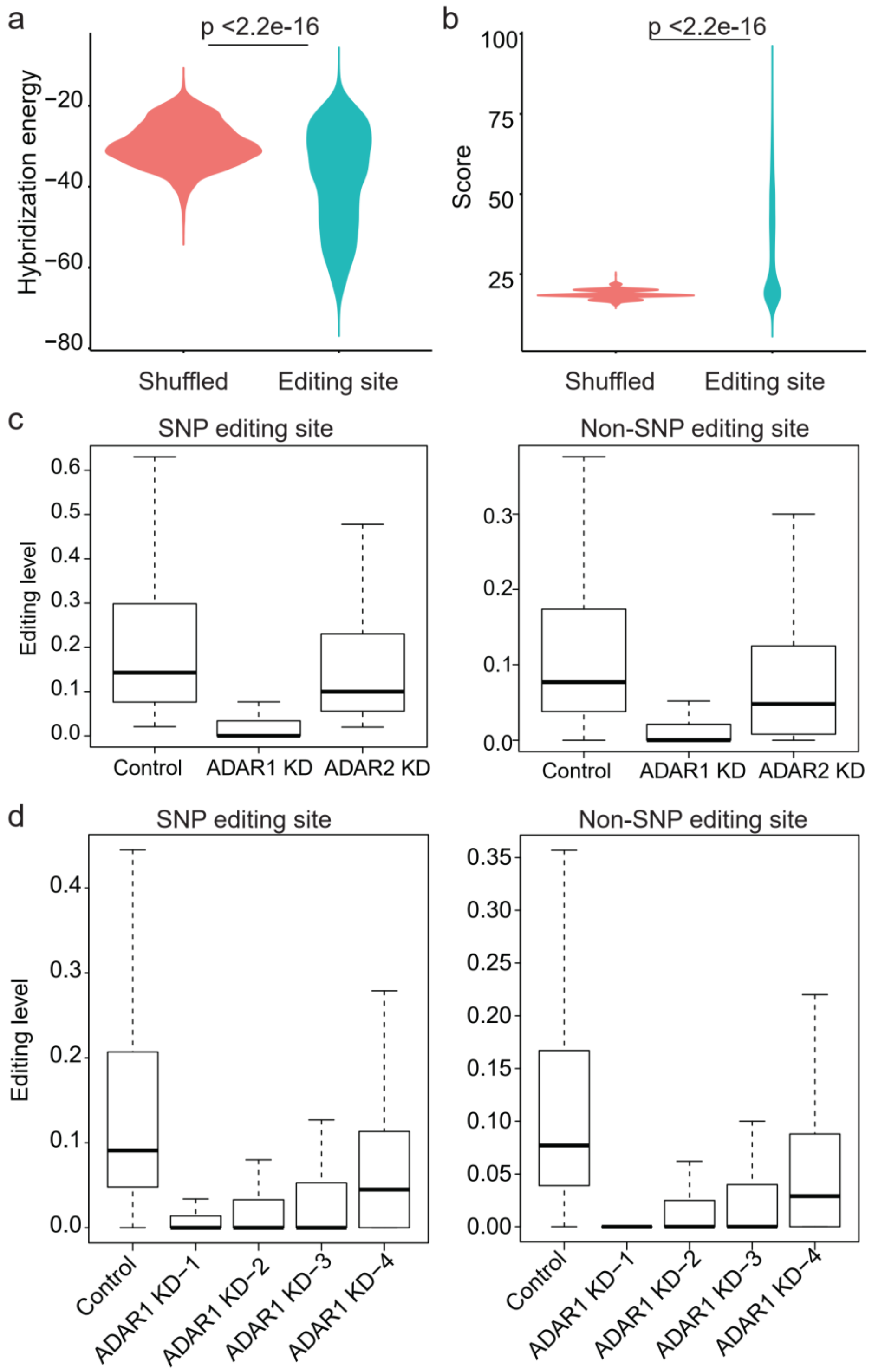

Fig. S4. The verification of SNP editing sites.

(**a**) Comparison of the hybridization energies between non-Alu SNP editing regions and their predicted complementary sequences with those between shuffled editing regions and their predicted complementary sequences. The analysis was performed as in **Fig. 1d**. About 15.2% of the SNP editing sites had a statistically significant ECS

(**Methods**).

(**b**) Comparison of the BLAST Scores between non-Alu SNP editing regions and shuffled editing regions. The analysis was performed as in **Fig. 1e**.

(**c**) Boxplots showing the editing level changes of SNP editing sites and non-SNP editing sites upon ADAR1 or ADAR2 knockdown in B cells (GM12004). Data were from Wang et al.

(**d**) Boxplots showing the editing level changes of SNP editing sites and non-SNP editing sites upon ADAR1 knockdown at different time points in B cells (GM12750). ADAR1 KD-1, ADAR1 KD-2, ADAR1 KD-3, and ADAR1 KD-4: 24h, 48h, 72h, and 96h after the siRNA transfection. Data were from Wang et al.

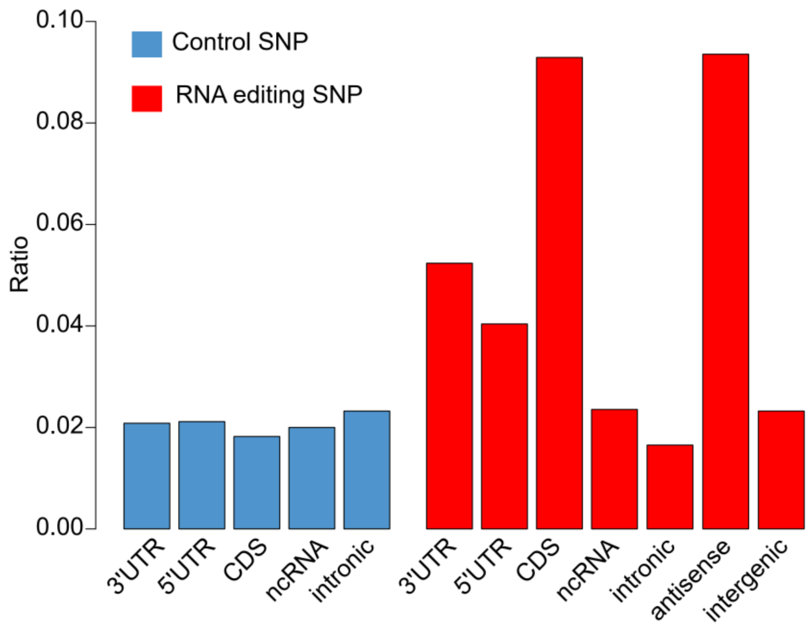

Fig. S5. Comparison of the ratios of RNA editing SNP and control SNP.

For RNA editing SNPs, the ratio was defined as the number of editing SNPs divided by the number of non-SNP editing sites. As a control, we calculated the ratio of control SNPs, which was defined as the number of SNPs with "A" as the reference allele divided by the total number of "A" in the given functional class. Notably, because the SNPs themselves had no strand information, we were unable to calculate the ratios of control SNPs in antisense transcripts and intergenic regions.

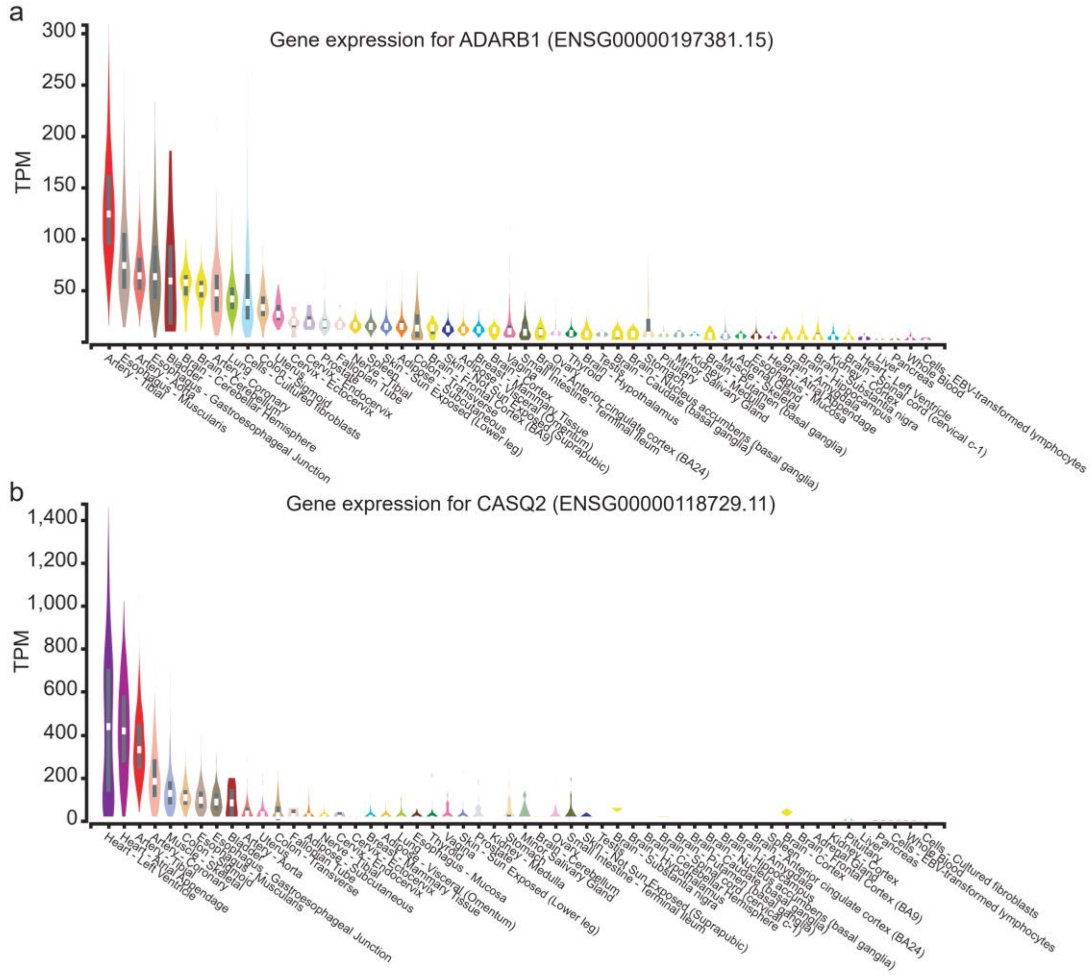

Fig. S6. ADAR2 and CASQ2 expression in different types of human tissues.

(**a**) ADAR2 and (**b**) CASQ2 expression profile in the GTEx dataset (v8). Data were from GTEx Portal.

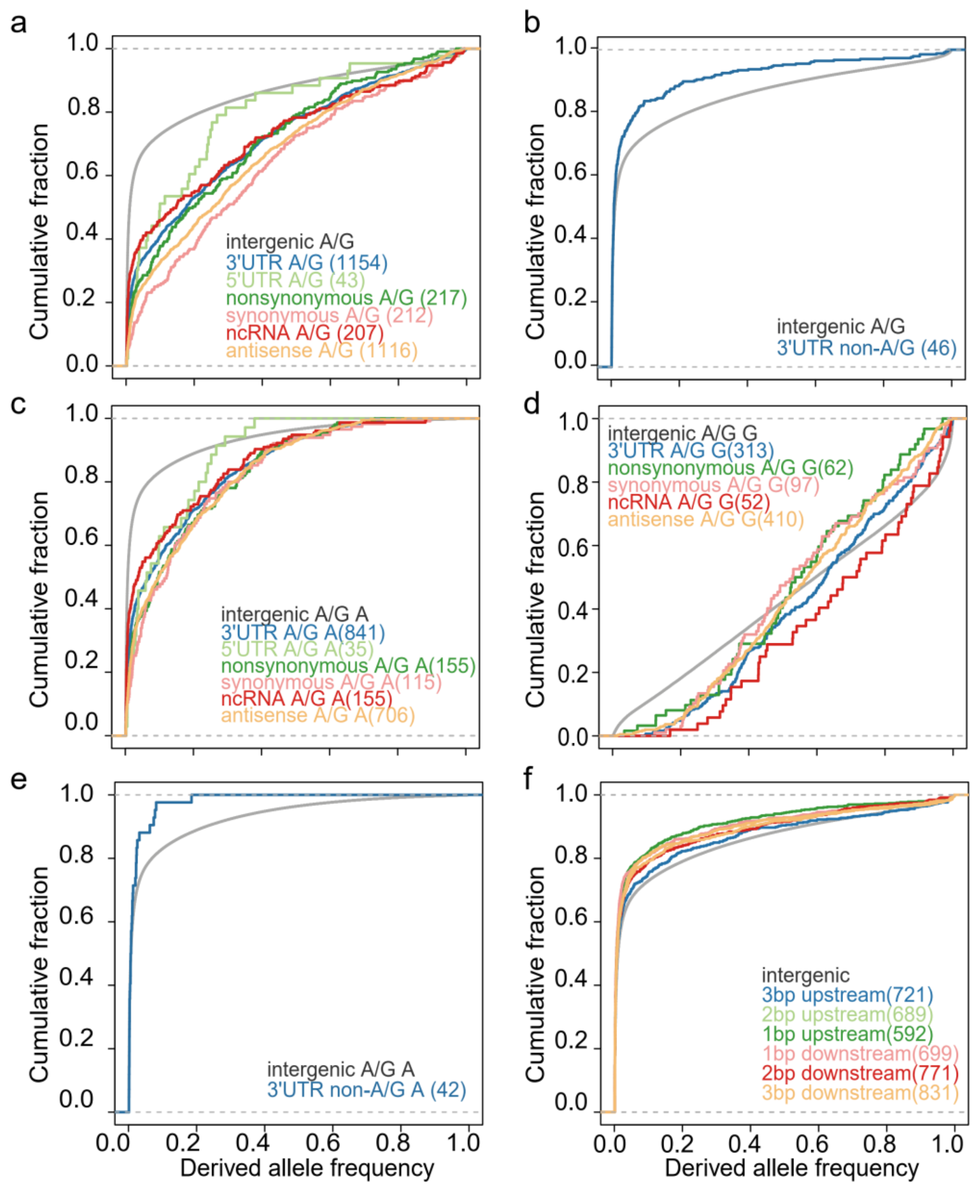

Fig. S7. The cumulative distribution of DAF.

(**a-b**) The cumulative distributions of DAF for A/G (**a**) or non-A/G (**b**) editing SNPs and intergenic control SNPs used in **Fig. 4a-b**.

(**c-d**) The cumulative distributions of DAF for A/G editing SNPs with A (**c**) or G (**d**) allele as the ancestral allele and intergenic control SNPs used in **Fig. 4c-d**.

(**e**) The cumulative distributions of DAF for non-A/G editing SNPs and intergenic control SNPs used in **Fig. 4e**.

(**f**) The cumulative distributions of DAF for the SNPs located in the upstream and downstream of 3' UTR non-SNP editing sites and intergenic control SNPs used in **Fig. 4f**.

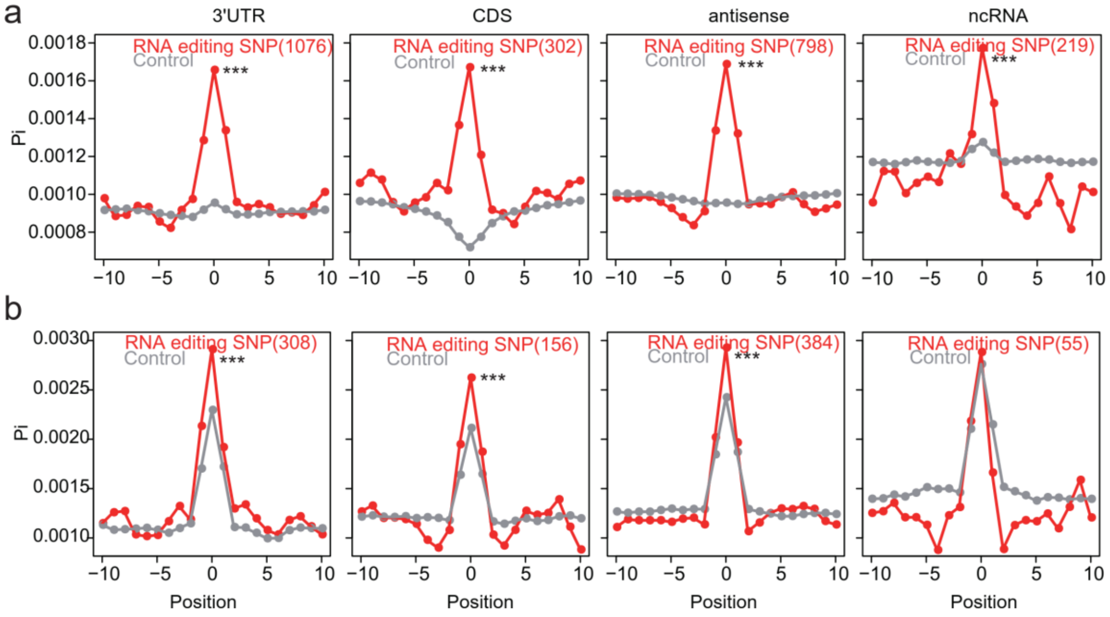

Fig. S8. Sliding window analysis of pi for A/G editing SNPs.

Editing SNPs with A or G allele as the ancestral allele were analyzed, respectively. A 200 bp sliding window (step size = 100nt) was used and the average pi in each window was shown. Moreover, a comparison between the A/G editing SNP containing window and the control A/G SNP containing window was performed. P values were calculated using the Mann-Whitney U test. ***, $p < 0.001$.

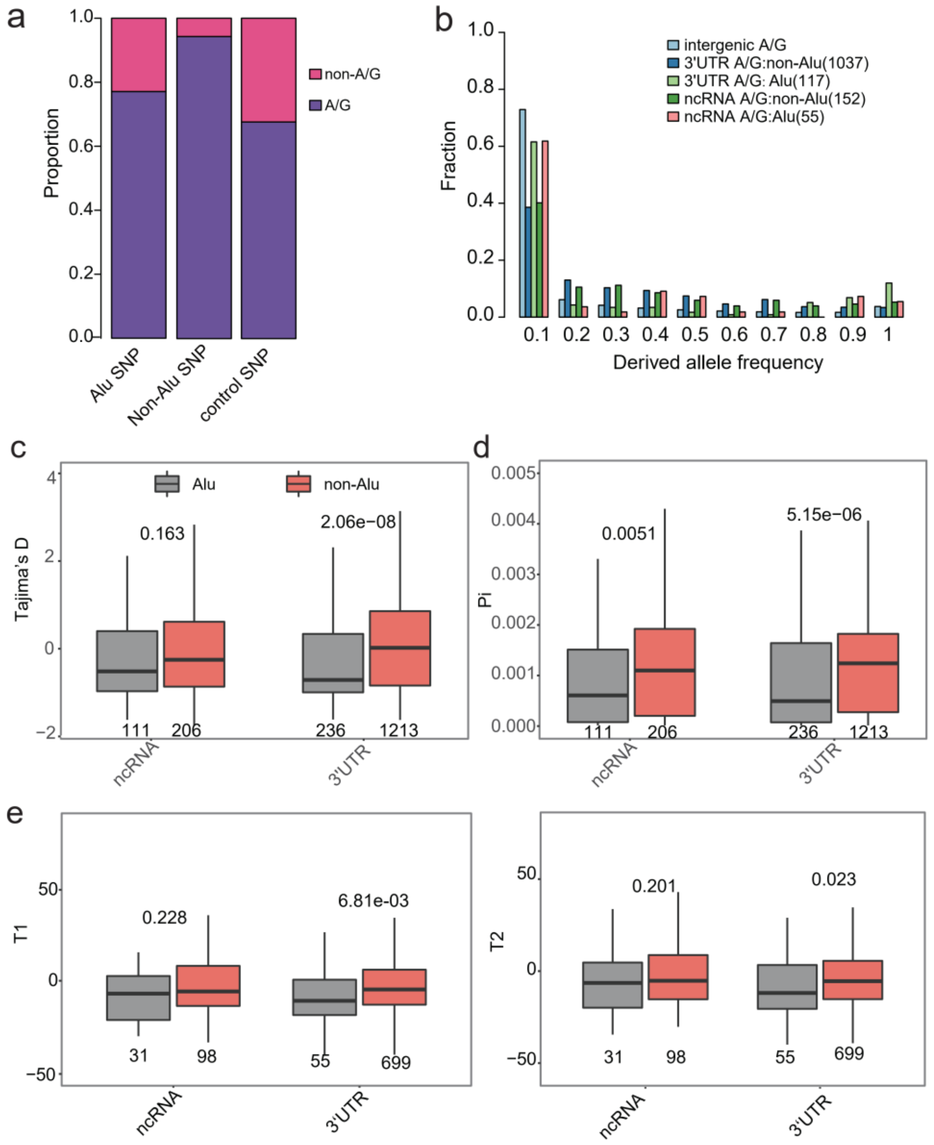

Fig. S9. non-Alu editing SNPs are subject to a stronger balancing selection compared with Alu editing SNPs.

(**a**) Comparison of the SNP types between Alu and non-Alu A/G editing SNPs.

(**b**) DAF distributions of A/G RNA editing SNPs. Non-Alu and Alu SNPs were analyzed, separately. Only 3'UTR and ncRNA sites were analyzed because of the limited number of CDS and 5'UTR Alu SNPs identified.

(**c-d**) Comparison of Tajima's D (**c**) and pi (**d**) between Alu and non-Alu A/G editing SNPs. P values were calculated using the Mann-Whitney U test.

(**e**) Comparison of T1 and T2 scores between Alu and non-Alu A/G editing SNPs. P values were calculated using the Mann-Whitney U test.

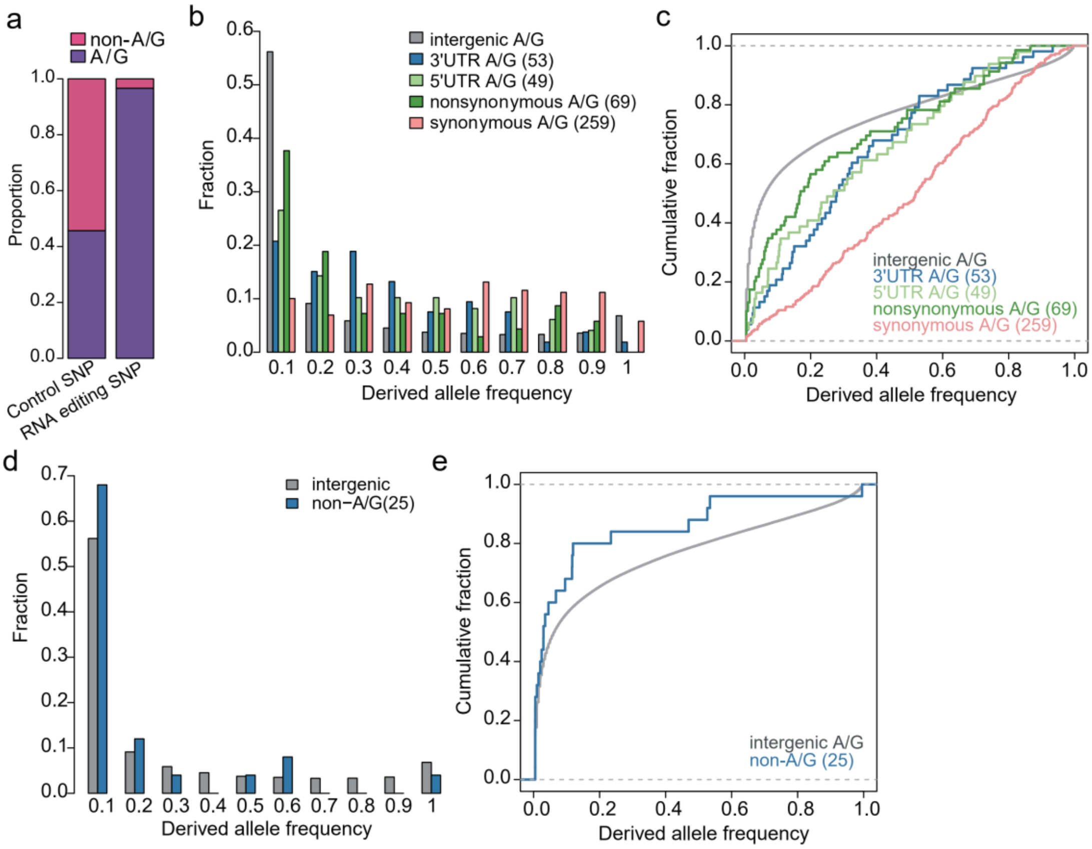

Fig. S10. DAF analysis reveals RNA editing SNPs as the target of balancing selection in flies.

(**a**) Comparison of the SNP types between editing SNPs and control SNPs. All SNPs in the DGRP dataset that are with A or T as the reference allele were selected as control SNPs.

(**b-c**) DAF distributions and cumulative distributions of DAF for A/G editing SNPs and intergenic control SNPs. P values were calculated with the Kolmogorov-Smirnov test by comparing the DAF distribution of editing SNPs in a defined genic location with the distribution of SNPs in intergenic regions (**Table S3**). The numbers of RNA editing SNPs in each genic location are shown in parentheses. Intergenic-A/G: all A/G SNPs located in the intergenic regions.

(**d-e**) DAF distributions and cumulative distributions of DAF for non-A/G editing SNPs and intergenic control SNPs. Because of the limited number of non-A/G editing SNPs, all SNPs were combined for analysis. Intergenic-non-A/G: all non-A/G SNPs located in the intergenic regions.
